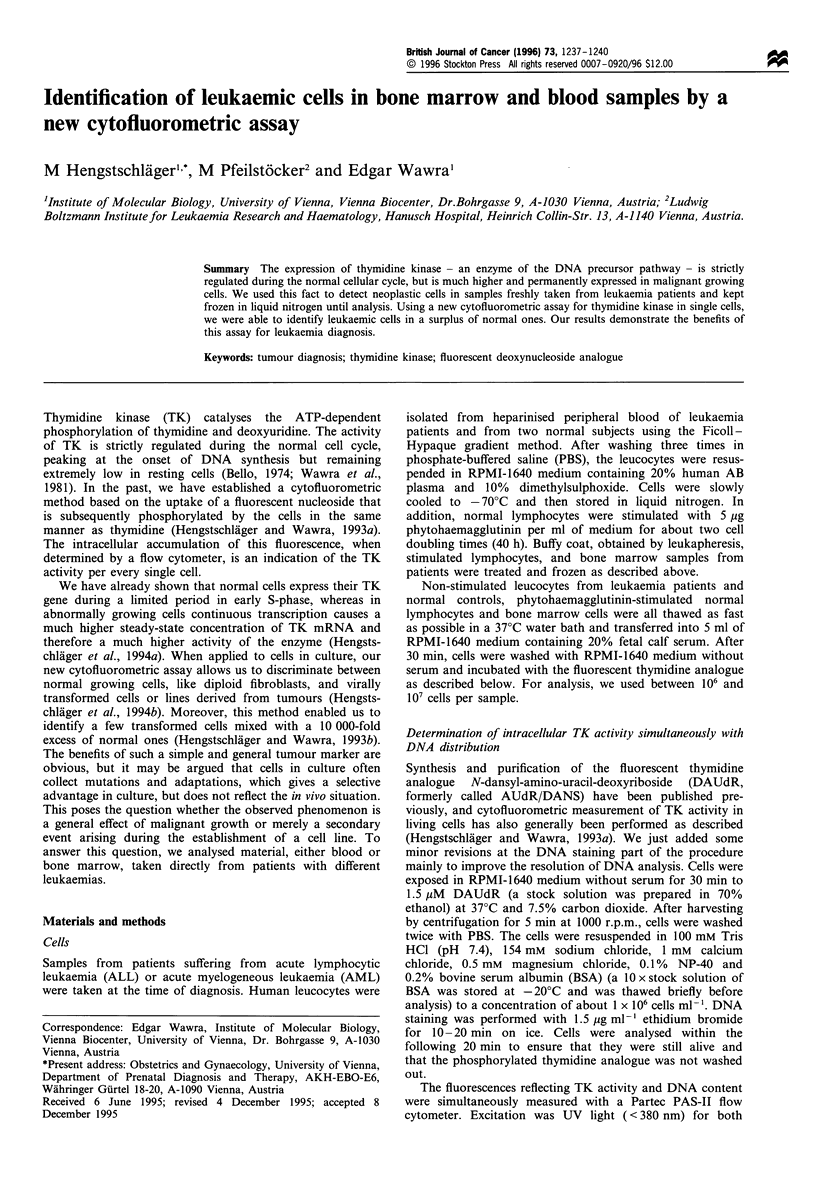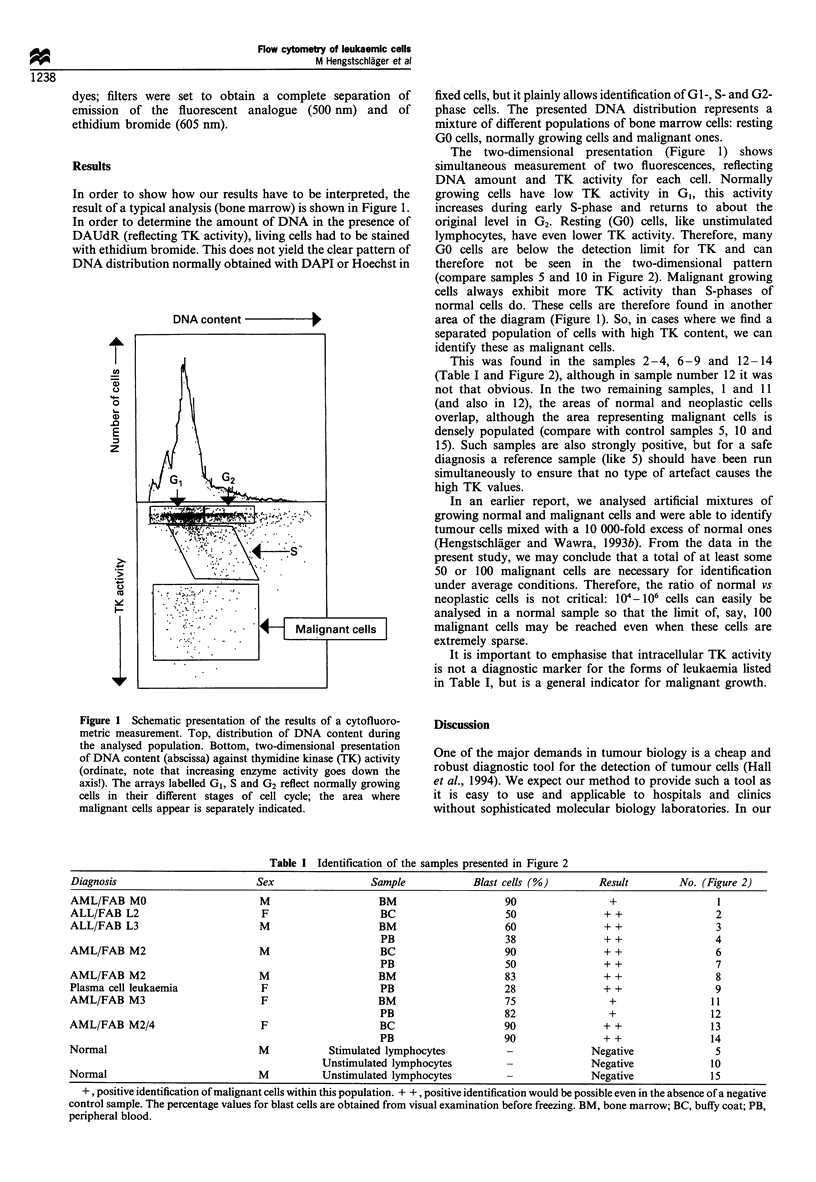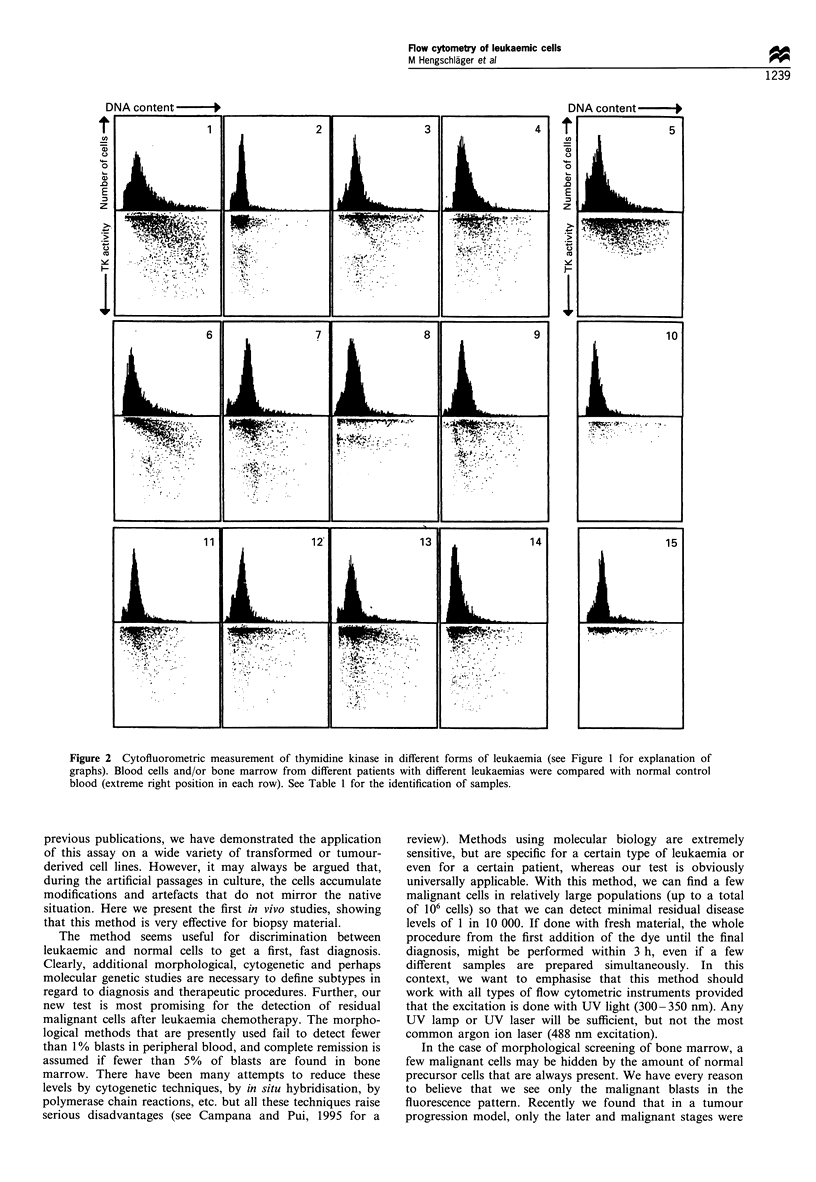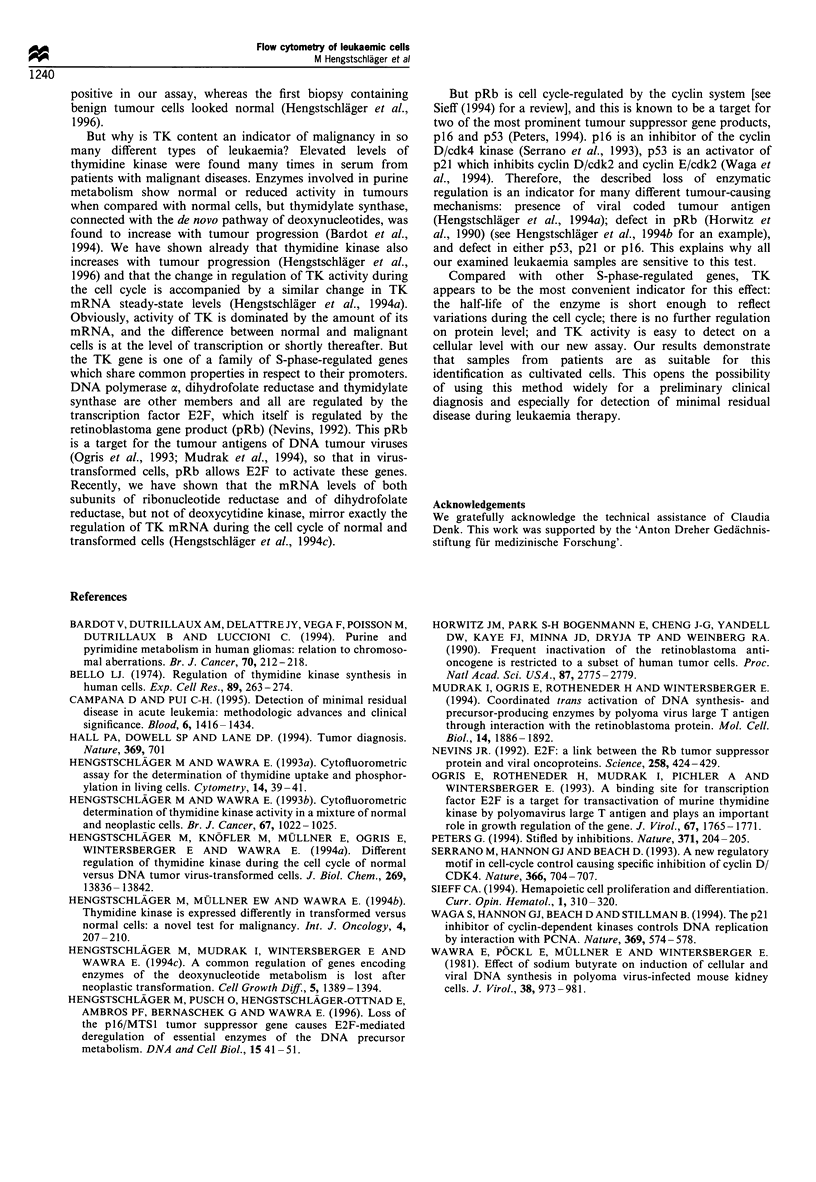# Identification of leukaemic cells in bone marrow and blood samples by a new cytofluorometric assay.

**DOI:** 10.1038/bjc.1996.237

**Published:** 1996-05

**Authors:** M. Hengstschläger, M. Pfeilstöcker, E. Wawra

**Affiliations:** Institute of Molecular Biology, University of Vienna, Vienna Biocenter, Austria.

## Abstract

The expression of thymidine kinase--an enzyme of the DNA precursor pathway--is strictly regulated during the normal cellular cycle, but is much higher and permanently expressed in malignant growing cells. We used this fact to detect neoplastic cells in samples freshly taken from leukaemia patients and kept frozen in liquid nitrogen until analysis. Using a new cytofluorometric assay for thymidine kinase in single cells, we were able to identify leukaemic cells in a surplus of normal ones. Our results demonstrate the benefits of this assay for leukaemia diagnosis.


British Journal of Cancer (1996) 73, 1237-1240
© 1996 Stockton Press All rights reserved 0007-0920/96 $12.00

### Identification of leukaemic cells in bone marrow and blood samples by a new cytofluorometric assay

M Hengstschläger[1,*], M Pfeilstöcker[2] and Edgar Wawra[1]

[1]*Institute of Molecular Biology, University of Vienna, Vienna Biocenter, Dr.Bohrgasse 9, A-1030 Vienna, Austria;* [2]*Ludwig Boltzmann Institute for Leukaemia Research and Haematology, Hanusch Hospital, Heinrich Collin-Str. 13, A-1140 Vienna, Austria.*

**Summary** The expression of thymidine kinase – an enzyme of the DNA precursor pathway – is strictly regulated during the normal cellular cycle, but is much higher and permanently expressed in malignant growing cells. We used this fact to detect neoplastic cells in samples freshly taken from leukaemia patients and kept frozen in liquid nitrogen until analysis. Using a new cytofluorometric assay for thymidine kinase in single cells, we were able to identify leukaemic cells in a surplus of normal ones. Our results demonstrate the benefits of this assay for leukaemia diagnosis.

**Keywords:** tumour diagnosis; thymidine kinase; fluorescent deoxynucleoside analogue

Thymidine kinase (TK) catalyses the ATP-dependent phosphorylation of thymidine and deoxyuridine. The activity of TK is strictly regulated during the normal cell cycle, peaking at the onset of DNA synthesis but remaining extremely low in resting cells (Bello, 1974; Wawra *et al.*, 1981). In the past, we have established a cytofluorometric method based on the uptake of a fluorescent nucleoside that is subsequently phosphorylated by the cells in the same manner as thymidine (Hengstschläger and Wawra, 1993*a*). The intracellular accumulation of this fluorescence, when determined by a flow cytometer, is an indication of the TK activity per every single cell.

We have already shown that normal cells express their TK gene during a limited period in early S-phase, whereas in abnormally growing cells continuous transcription causes a much higher steady-state concentration of TK mRNA and therefore a much higher activity of the enzyme (Hengstschläger *et al.*, 1994*a*). When applied to cells in culture, our new cytofluorometric assay allows us to discriminate between normal growing cells, like diploid fibroblasts, and virally transformed cells or lines derived from tumours (Hengstschläger *et al.*, 1994*b*). Moreover, this method enabled us to identify a few transformed cells mixed with a 10 000-fold excess of normal ones (Hengstschläger and Wawra, 1993*b*). The benefits of such a simple and general tumour marker are obvious, but it may be argued that cells in culture often collect mutations and adaptations, which gives a selective advantage in culture, but does not reflect the *in vivo* situation. This poses the question whether the observed phenomenon is a general effect of malignant growth or merely a secondary event arising during the establishment of a cell line. To answer this question, we analysed material, either blood or bone marrow, taken directly from patients with different leukaemias.

### Materials and methods

### *Cells*

Samples from patients suffering from acute lymphocytic leukaemia (ALL) or acute myelogeneous leukaemia (AML) were taken at the time of diagnosis. Human leucocytes were isolated from heparinised peripheral blood of leukaemia patients and from two normal subjects using the Ficoll–Hypaque gradient method. After washing three times in phosphate-buffered saline (PBS), the leucocytes were resuspended in RPMI-1640 medium containing 20% human AB plasma and 10% dimethylsulphoxide. Cells were slowly cooled to −70°C and then stored in liquid nitrogen. In addition, normal lymphocytes were stimulated with 5 $\mu$g phytohaemagglutinin per ml of medium for about two cell doubling times (40 h). Buffy coat, obtained by leukapheresis, stimulated lymphocytes, and bone marrow samples from patients were treated and frozen as described above.

Non-stimulated leucocytes from leukaemia patients and normal controls, phytohaemagglutinin-stimulated normal lymphocytes and bone marrow cells were all thawed as fast as possible in a 37°C water bath and transferred into 5 ml of RPMI-1640 medium containing 20% fetal calf serum. After 30 min, cells were washed with RPMI-1640 medium without serum and incubated with the fluorescent thymidine analogue as described below. For analysis, we used between $10^6$ and $10^7$ cells per sample.

### *Determination of intracellular TK activity simultaneously with DNA distribution*

Synthesis and purification of the fluorescent thymidine analogue *N*-dansyl-amino-uracil-deoxyriboside (DAUdR, formerly called AUdR/DANS) have been published previously, and cytofluorometric measurement of TK activity in living cells has also generally been performed as described (Hengstschläger and Wawra, 1993*a*). We just added some minor revisions at the DNA staining part of the procedure mainly to improve the resolution of DNA analysis. Cells were exposed in RPMI-1640 medium without serum for 30 min to 1.5 $\mu$M DAUdR (a stock solution was prepared in 70% ethanol) at 37°C and 7.5% carbon dioxide. After harvesting by centrifugation for 5 min at 1000 r.p.m., cells were washed twice with PBS. The cells were resuspended in 100 mM Tris HCl (pH 7.4), 154 mM sodium chloride, 1 mM calcium chloride, 0.5 mM magnesium chloride, 0.1% NP-40 and 0.2% bovine serum albumin (BSA) (a 10 × stock solution of BSA was stored at −20°C and was thawed briefly before analysis) to a concentration of about $1 \times 10^6$ cells $ml^{-1}$. DNA staining was performed with 1.5 $\mu g\ ml^{-1}$ ethidium bromide for 10–20 min on ice. Cells were analysed within the following 20 min to ensure that they were still alive and that the phosphorylated thymidine analogue was not washed out.

The fluorescences reflecting TK activity and DNA content were simultaneously measured with a Partec PAS-II flow cytometer. Excitation was UV light (<380 nm) for both

Correspondence: Edgar Wawra, Institute of Molecular Biology, Vienna Biocenter, University of Vienna, Dr. Bohrgasse 9, A-1030 Vienna, Austria
*Present address: Obstetrics and Gynaecology, University of Vienna, Department of Prenatal Diagnosis and Therapy, AKH-EBO-E6, Währinger Gürtel 18-20, A-1090 Vienna, Austria
Received 6 June 1995; revised 4 December 1995; accepted 8 December 1995

dyes; filters were set to obtain a complete separation of emission of the fluorescent analogue (500 nm) and of ethidium bromide (605 nm).

### Results

In order to show how our results have to be interpreted, the result of a typical analysis (bone marrow) is shown in Figure 1. In order to determine the amount of DNA in the presence of DAUdR (reflecting TK activity), living cells had to be stained with ethidium bromide. This does not yield the clear pattern of DNA distribution normally obtained with DAPI or Hoechst in fixed cells, but it plainly allows identification of G1-, S- and G2-phase cells. The presented DNA distribution represents a mixture of different populations of bone marrow cells: resting G0 cells, normally growing cells and malignant ones.

Figure 1 Schematic presentation of the results of a cytofluorometric measurement. Top, distribution of DNA content during the analysed population. Bottom, two-dimensional presentation of DNA content (abscissa) against thymidine kinase (TK) activity (ordinate, note that increasing enzyme activity goes down the axis!). The arrays labelled $G_1$, S and $G_2$ reflect normally growing cells in their different stages of cell cycle; the area where malignant cells appear is separately indicated.

The two-dimensional presentation (Figure 1) shows simultaneous measurement of two fluorescences, reflecting DNA amount and TK activity for each cell. Normally growing cells have low TK activity in $G_1$, this activity increases during early S-phase and returns to about the original level in $G_2$. Resting (G0) cells, like unstimulated lymphocytes, have even lower TK activity. Therefore, many G0 cells are below the detection limit for TK and can therefore not be seen in the two-dimensional pattern (compare samples 5 and 10 in Figure 2). Malignant growing cells always exhibit more TK activity than S-phases of normal cells do. These cells are therefore found in another area of the diagram (Figure 1). So, in cases where we find a separated population of cells with high TK content, we can identify these as malignant cells.

This was found in the samples 2–4, 6–9 and 12–14 (Table I and Figure 2), although in sample number 12 it was not that obvious. In the two remaining samples, 1 and 11 (and also in 12), the areas of normal and neoplastic cells overlap, although the area representing malignant cells is densely populated (compare with control samples 5, 10 and 15). Such samples are also strongly positive, but for a safe diagnosis a reference sample (like 5) should have been run simultaneously to ensure that no type of artefact causes the high TK values.

In an earlier report, we analysed artificial mixtures of growing normal and malignant cells and were able to identify tumour cells mixed with a 10 000-fold excess of normal ones (Hengstschläger and Wawra, 1993*b*). From the data in the present study, we may conclude that a total of at least some 50 or 100 malignant cells are necessary for identification under average conditions. Therefore, the ratio of normal *vs* neoplastic cells is not critical: $10^4$–$10^6$ cells can easily be analysed in a normal sample so that the limit of, say, 100 malignant cells may be reached even when these cells are extremely sparse.

It is important to emphasise that intracellular TK activity is not a diagnostic marker for the forms of leukaemia listed in Table I, but is a general indicator for malignant growth.

### Discussion

One of the major demands in tumour biology is a cheap and robust diagnostic tool for the detection of tumour cells (Hall *et al.*, 1994). We expect our method to provide such a tool as it is easy to use and applicable to hospitals and clinics without sophisticated molecular biology laboratories. In our

**Table I** Identification of the samples presented in Figure 2

| *Diagnosis* | *Sex* | *Sample* | *Blast cells (%)* | *Result* | *No. (Figure 2)* |
|---|---|---|---|---|---|
| AML/FAB M0 | M | BM | 90 | + | 1 |
| ALL/FAB L2 | F | BC | 50 | + + | 2 |
| ALL/FAB L3 | M | BM | 60 | + + | 3 |
| | | PB | 38 | + + | 4 |
| AML/FAB M2 | M | BC | 90 | + + | 6 |
| | | PB | 50 | + + | 7 |
| AML/FAB M2 | M | BM | 83 | + + | 8 |
| Plasma cell leukaemia | F | PB | 28 | + + | 9 |
| AML/FAB M3 | F | BM | 75 | + | 11 |
| | | PB | 82 | + | 12 |
| AML/FAB M2/4 | F | BC | 90 | + + | 13 |
| | | PB | 90 | + + | 14 |
| Normal | M | Stimulated lymphocytes | – | Negative | 5 |
| | | Unstimulated lymphocytes | – | Negative | 10 |
| Normal | M | Unstimulated lymphocytes | – | Negative | 15 |

+, positive identification of malignant cells within this population. + +, positive identification would be possible even in the absence of a negative control sample. The percentage values for blast cells are obtained from visual examination before freezing. BM, bone marrow; BC, buffy coat; PB, peripheral blood.

**Figure 2** Cytofluorometric measurement of thymidine kinase in different forms of leukaemia (see Figure 1 for explanation of graphs). Blood cells and/or bone marrow from different patients with different leukaemias were compared with normal control blood (extreme right position in each row). See Table 1 for the identification of samples.

previous publications, we have demonstrated the application of this assay on a wide variety of transformed or tumour-derived cell lines. However, it may always be argued that, during the artificial passages in culture, the cells accumulate modifications and artefacts that do not mirror the native situation. Here we present the first *in vivo* studies, showing that this method is very effective for biopsy material.

The method seems useful for discrimination between leukaemic and normal cells to get a first, fast diagnosis. Clearly, additional morphological, cytogenetic and perhaps molecular genetic studies are necessary to define subtypes in regard to diagnosis and therapeutic procedures. Further, our new test is most promising for the detection of residual malignant cells after leukaemia chemotherapy. The morphological methods that are presently used fail to detect fewer than 1% blasts in peripheral blood, and complete remission is assumed if fewer than 5% of blasts are found in bone marrow. There have been many attempts to reduce these levels by cytogenetic techniques, by *in situ* hybridisation, by polymerase chain reactions, etc. but all these techniques raise serious disadvantages (see Campana and Pui, 1995 for a review). Methods using molecular biology are extremely sensitive, but are specific for a certain type of leukaemia or even for a certain patient, whereas our test is obviously universally applicable. With this method, we can find a few malignant cells in relatively large populations (up to a total of $10^6$ cells) so that we can detect minimal residual disease levels of 1 in 10 000. If done with fresh material, the whole procedure from the first addition of the dye until the final diagnosis, might be performed within 3 h, even if a few different samples are prepared simultaneously. In this context, we want to emphasise that this method should work with all types of flow cytometric instruments provided that the excitation is done with UV light (300–350 nm). Any UV lamp or UV laser will be sufficient, but not the most common argon ion laser (488 nm excitation).

In the case of morphological screening of bone marrow, a few malignant cells may be hidden by the amount of normal precursor cells that are always present. We have every reason to believe that we see only the malignant blasts in the fluorescence pattern. Recently we found that in a tumour progression model, only the later and malignant stages were positive in our assay, whereas the first biopsy containing benign tumour cells looked normal (Hengstschläger *et al.*, 1996).

But why is TK content an indicator of malignancy in so many different types of leukaemia? Elevated levels of thymidine kinase were found many times in serum from patients with malignant diseases. Enzymes involved in purine metabolism show normal or reduced activity in tumours when compared with normal cells, but thymidylate synthase, connected with the *de novo* pathway of deoxynucleotides, was found to increase with tumour progression (Bardot *et al.*, 1994). We have shown already that thymidine kinase also increases with tumour progression (Hengstschläger *et al.*, 1996) and that the change in regulation of TK activity during the cell cycle is accompanied by a similar change in TK mRNA steady-state levels (Hengstschläger *et al.*, 1994*a*). Obviously, activity of TK is dominated by the amount of its mRNA, and the difference between normal and malignant cells is at the level of transcription or shortly thereafter. But the TK gene is one of a family of S-phase-regulated genes which share common properties in respect to their promoters. DNA polymerase $\alpha$, dihydrofolate reductase and thymidylate synthase are other members and all are regulated by the transcription factor E2F, which itself is regulated by the retinoblastoma gene product (pRb) (Nevins, 1992). This pRb is a target for the tumour antigens of DNA tumour viruses (Ogris *et al.*, 1993; Mudrak *et al.*, 1994), so that in virus-transformed cells, pRb allows E2F to activate these genes. Recently, we have shown that the mRNA levels of both subunits of ribonucleotide reductase and of dihydrofolate reductase, but not of deoxycytidine kinase, mirror exactly the regulation of TK mRNA during the cell cycle of normal and transformed cells (Hengstschläger *et al.*, 1994*c*).

But pRb is cell cycle-regulated by the cyclin system [see Sieff (1994) for a review], and this is known to be a target for two of the most prominent tumour suppressor gene products, p16 and p53 (Peters, 1994). p16 is an inhibitor of the cyclin D/cdk4 kinase (Serrano *et al.*, 1993), p53 is an activator of p21 which inhibits cyclin D/cdk2 and cyclin E/cdk2 (Waga *et al.*, 1994). Therefore, the described loss of enzymatic regulation is an indicator for many different tumour-causing mechanisms: presence of viral coded tumour antigen (Hengstschläger *et al.*, 1994*a*); defect in pRb (Horwitz *et al.*, 1990) (see Hengstschläger *et al.*, 1994*b* for an example), and defect in either p53, p21 or p16. This explains why all our examined leukaemia samples are sensitive to this test.

Compared with other S-phase-regulated genes, TK appears to be the most convenient indicator for this effect: the half-life of the enzyme is short enough to reflect variations during the cell cycle; there is no further regulation on protein level; and TK activity is easy to detect on a cellular level with our new assay. Our results demonstrate that samples from patients are as suitable for this identification as cultivated cells. This opens the possibility of using this method widely for a preliminary clinical diagnosis and especially for detection of minimal residual disease during leukaemia therapy.

**Acknowledgements**
We gratefully acknowledge the technical assistance of Claudia Denk. This work was supported by the 'Anton Dreher Gedächnisstiftung für medizinische Forschung'.

### References

BARDOT V, DUTRILLAUX AM, DELATTRE JY, VEGA F, POISSON M, DUTRILLAUX B AND LUCCIONI C. (1994). Purine and pyrimidine metabolism in human gliomas: relation to chromosomal aberrations. *Br. J. Cancer*, **70**, 212–218.

BELLO LJ. (1974). Regulation of thymidine kinase synthesis in human cells. *Exp. Cell Res.*, **89**, 263–274.

CAMPANA D AND PUI C-H. (1995). Detection of minimal residual disease in acute leukemia: methodologic advances and clinical significance. *Blood*, **6**, 1416–1434.

HALL PA, DOWELL SP AND LANE DP. (1994). Tumor diagnosis. *Nature*, **369**, 701

HENGSTSCHLÄGER M AND WAWRA E. (1993*a*). Cytofluorometric assay for the determination of thymidine uptake and phosphorylation in living cells. *Cytometry*, **14**, 39–41.

HENGSTSCHLÄGER M AND WAWRA E. (1993*b*). Cytofluorometric determination of thymidine kinase activity in a mixture of normal and neoplastic cells. *Br. J. Cancer*, **67**, 1022–1025.

HENGSTSCHLÄGER M, KNÖFLER M, MÜLLNER E, OGRIS E, WINTERSBERGER E AND WAWRA E. (1994*a*). Different regulation of thymidine kinase during the cell cycle of normal versus DNA tumor virus-transformed cells. *J. Biol. Chem.*, **269**, 13836–13842.

HENGSTSCHLÄGER M, MÜLLNER EW AND WAWRA E. (1994*b*). Thymidine kinase is expressed differently in transformed versus normal cells: a novel test for malignancy. *Int. J. Oncology*, **4**, 207–210.

HENGSTSCHLÄGER M, MUDRAK I, WINTERSBERGER E AND WAWRA E. (1994*c*). A common regulation of genes encoding enzymes of the deoxynucleotide metabolism is lost after neoplastic transformation. *Cell Growth Diff.*, **5**, 1389–1394.

HENGSTSCHLÄGER M, PUSCH O, HENGSTSCHLÄGER-OTTNAD E, AMBROS PF, BERNASCHEK G AND WAWRA E. (1996). Loss of the p16/MTS1 tumor suppressor gene causes E2F-mediated deregulation of essential enzymes of the DNA precursor metabolism. *DNA and Cell Biol.*, **15** 41–51.

HORWITZ JM, PARK S-H BOGENMANN E, CHENG J-G, YANDELL DW, KAYE FJ, MINNA JD, DRYJA TP AND WEINBERG RA. (1990). Frequent inactivation of the retinoblastoma anti-oncogene is restricted to a subset of human tumor cells. *Proc. Natl Acad. Sci. USA.*, **87**, 2775–2779.

MUDRAK I, OGRIS E, ROTHENEDER H AND WINTERSBERGER E. (1994). Coordinated *trans* activation of DNA synthesis- and precursor-producing enzymes by polyoma virus large T antigen through interaction with the retinoblastoma protein. *Mol. Cell. Biol.*, **14**, 1886–1892.

NEVINS JR. (1992). E2F: a link between the Rb tumor suppressor protein and viral oncoproteins. *Science*, **258**, 424–429.

OGRIS E, ROTHENEDER H, MUDRAK I, PICHLER A AND WINTERSBERGER E. (1993). A binding site for transcription factor E2F is a target for transactivation of murine thymidine kinase by polyomavirus large T antigen and plays an important role in growth regulation of the gene. *J. Virol.*, **67**, 1765–1771.

PETERS G. (1994). Stifled by inhibitions. *Nature*, **371**, 204–205.

SERRANO M, HANNON GJ AND BEACH D. (1993). A new regulatory motif in cell-cycle control causing specific inhibition of cyclin D/CDK4. *Nature*, **366**, 704–707.

SIEFF CA. (1994). Hemapoietic cell proliferation and differentiation. *Curr. Opin. Hematol.*, **1**, 310–320.

WAGA S, HANNON GJ, BEACH D AND STILLMAN B. (1994). The p21 inhibitor of cyclin-dependent kinases controls DNA replication by interaction with PCNA. *Nature*, **369**, 574–578.

WAWRA E, PÖCKL E, MÜLLNER E AND WINTERSBERGER E. (1981). Effect of sodium butyrate on induction of cellular and viral DNA synthesis in polyoma virus-infected mouse kidney cells. *J. Virol.*, **38**, 973–981.